# Coughing children in family practice and primary care: a systematic review of prevalence, aetiology and prognosis

**DOI:** 10.1186/s12887-021-02739-4

**Published:** 2021-06-04

**Authors:** Milena Bergmann, Jörg Haasenritter, Dominik Beidatsch, Sonja Schwarm, Kaja Hörner, Stefan Bösner, Paula Grevenrath, Laura Schmidt, Annika Viniol, Norbert Donner-Banzhoff, Annette Becker

**Affiliations:** Department of General Practice/Family Medicine, University of Marburg, Karl-von-Frisch-Str. 4, 35043 Marburg, Germany

**Keywords:** Cough, Children, Primary care, Prevalence, Prognosis, Aetiology, Symptom evaluation

## Abstract

**Background:**

For evidence-based decision making, primary care physicians need to have specific and reliable information on the pre-test probabilities of underlying diseases and a symptom’s course. We performed a systematic review of symptom-evaluating studies in primary care, following three research questions: (1) What is the prevalence of the symptom cough in children consulting primary care physicians? (2) What are the underlying aetiologies of cough and the respective frequencies? (3) What is the prognosis of children with cough?

**Methods:**

Following a pre-defined algorithm and independent double reviewer ratings we searched MEDLINE and EMBASE. All quantitative original research articles in English, French or German were included if they focused on unselected study populations of children consulting a primary care physician for cough. We used the random effects model for meta-analysis in subgroups, if justifiable in terms of heterogeneity.

**Results:**

We identified 14 eligible studies on prevalence, five on aetiology and one on prognosis. Prevalence estimates varied between 4.7 and 23.3% of all reasons for an encounter, or up to estimates of 60% when related to patients or consultations. Cough in children is more frequent than in adults, with lowest prevalences in adolescents and in summer. Acute cough is mostly caused by upper respiratory tract infections (62.4%) and bronchitis (33.3%); subacute or chronic cough by recurrent respiratory tract infection (27.7%), asthma (up to 50.4% in cough persisting more than 3 weeks), and pertussis (37.2%). Potentially serious diseases like croup, pneumonia or tuberculosis are scarce. In children with subacute and chronic cough the total duration of cough ranged from 24 to 192 days. About 62.3% of children suffering from prolonged cough are still coughing two months after the beginning of symptoms.

**Conclusion:**

Cough is one of the most frequent reasons for an encounter in primary care. Our findings fit in with current guideline recommendations supporting a thoughtful wait-and-see approach in acute cough and a special awareness in chronic cough of the possibility of asthma and pertussis. Further evidence of aetiological pre-test probabilities is needed to assess the diagnostic gain based on patient history and clinical signs for differential diagnoses of cough in children.

**Supplementary Information:**

The online version contains supplementary material available at 10.1186/s12887-021-02739-4.

## Background

Cough is a frequent reason for encounters for both children [1, 2] and adults [3] in ambulatory care. It often gives serious concern to parents [4, 5]. Especially when prolonged, cough impairs daily activities or sleep and children’s and caregivers’ quality of life [2, 6, 7]. Therefore, 30 to 40% of coughing children consult a physician [8].

General practitioners (GPs), family physicians or paediatricians triage self-limiting, prolonged, and potentially life-threatening courses. In this respect assumed or research-based pre-test probabilities and prognosis drive GPs’ decision making and action.

Current guideline recommendations are mainly based on secondary or tertiary care studies [9, 10], which do not necessarily conform with the situation in primary care. We therefore performed a systematic literature review, working on the following research questions (1) What is the prevalence of the symptom cough in children consulting primary care practices, or how often do children in general practice or paediatric practices consult for cough? (2) What are the underlying diagnoses and their respective frequencies? (3) What is the usual course of disease or what is the prognosis of these children?

## Methods

We performed a systematic review of symptom-evaluating studies. Based on the PRISMA statement [11] (Additional File 1) and the recommendation of Donner-Banzhoff [12] et al., methods were pre-specified in a protocol. Our working group applied the same methods on abdominal pain, tiredness, chest pain, dizziness and dyspnoea [13–17].

### Data sources and search strategy

We searched MEDLINE in June 2012, updated our search in 2019, and EMBASE in January 2015, updated in 2020. The reference lists all relevant papers were screened (snowball search). Our search was limited to publications in English, French, and German. The search syntax comprised the term “cough” in all possible wordings in title/abstract OR as MeSH Term, and the term “primary care” in all possible wordings in title/abstract OR in mailing address or in the name of the authors’ institute OR as MeSH Term OR a journal representing primary care research. For this we searched for general practice/family medicine, as well as paediatric primary care. For the entire search syntax see Additional File 2.

### Study selection and inclusion/exclusion criteria

We first screened titles and abstracts with respect to (1) original research article, (2) primary care as the study setting, and (3) “cough” as reason for encounter (primary or secondary reason for consultation).

The full text publications were assessed for our inclusion criteria as above plus (4) an unselected study population regarding the likelihood of the underlying aetiology, and (5) data available on incidence, prevalence, underlying diagnoses or prognosis of cough. All criteria had to be fulfilled. We excluded qualitative studies, case reports, reviews, studies without available full text, and studies recruiting in secondary or tertiary care, emergency departments/out-of-hours-services or population-based settings. No studies in which patients were systematically asked about cough were included. To avoid pre-selection, we did not consider studies that excluded patients with chronic diseases, studies, which recruited patients with an increased probability for a particular diagnosis or with cough being part of a required symptom combination (e.g. cough plus fever or expectoration). We included only studies on children. Reasons for exclusion were documented. The selection process was performed by two independently working reviewers: MB/DB or MB/SS (except the search updates 2019/2020). In case of disagreement, reviewers discussed their ratings or, secondly, consulted a third reviewer (AB).

### Data extraction

For each publication, we extracted bibliographic information (author, publication year, title, journal), country, inclusion/exclusion criteria, definition of cough, characteristics of physicians and practices, type of recruitment, information on study population (sample size, age, gender distribution) and study duration.

For prevalence/incidence data, we extracted the number of cough cases and the number and type of the reference study sample. For aetiology we registered all diagnostic categories with their relative and absolute frequencies, and we extracted any kind of prognostic data. We analysed all available publications of each study, and in doubt contacted the authors personally (*n* = 7).

### Assessment of methodical quality and risk of bias

Our working group developed a literature-based tool for evaluating risk of bias and clinical heterogeneity in symptom studies [12, 18]. A validation study is still running. Two reviewers (KH, MB) independently assessed 16 items in four key domains (Additional File 3) and rated the risk of bias in patient selection, data collection/patient flow, and in diagnostic and prognostic work-up. The risk of substantial variation/clinical heterogeneity was judged.

#### Statistics

The proportions of prevalence/incidence and underlying aetiologies plus 95% confidence intervals were calculated. Study outcomes vary in nominators and denominators of the frequency measure. For example, some counted consultations for cough in relation to all consultations, while others referred to reasons for encounters or all patients consulting a physician within a certain time frame. Since this had substantial impact on the results, we grouped the identified studies by these pre-specified denominators. Furthermore, we did subgrouping by duration of cough (pre-specified) and regional characteristics (post hoc). Aetiological and prognostic outcomes were analysed descriptively. Probability estimates and variation between studies are visualized with forest plots. For meta-analysis, we used the random-effects model (for distribution across studies) [19].

Study outcomes vary due to methodological (study design and bias) and clinical heterogeneity (study population, inclusion criteria, healthcare system, diagnostic work-up) [19]. We used χ^2^, *p*-value and I^2^: A heterogeneity beyond chance is characterized by high values of χ^2^ and low *p*-values; the portion of variability that is not due to chance is marked by I^2^ [19].

We used the software R (R Foundation for statistical Computing, Vienna, Austria, version 3.4.4) and RStudio V (RStudio, Inc., version 1.1.442).

## Results

**Fig. 1:**
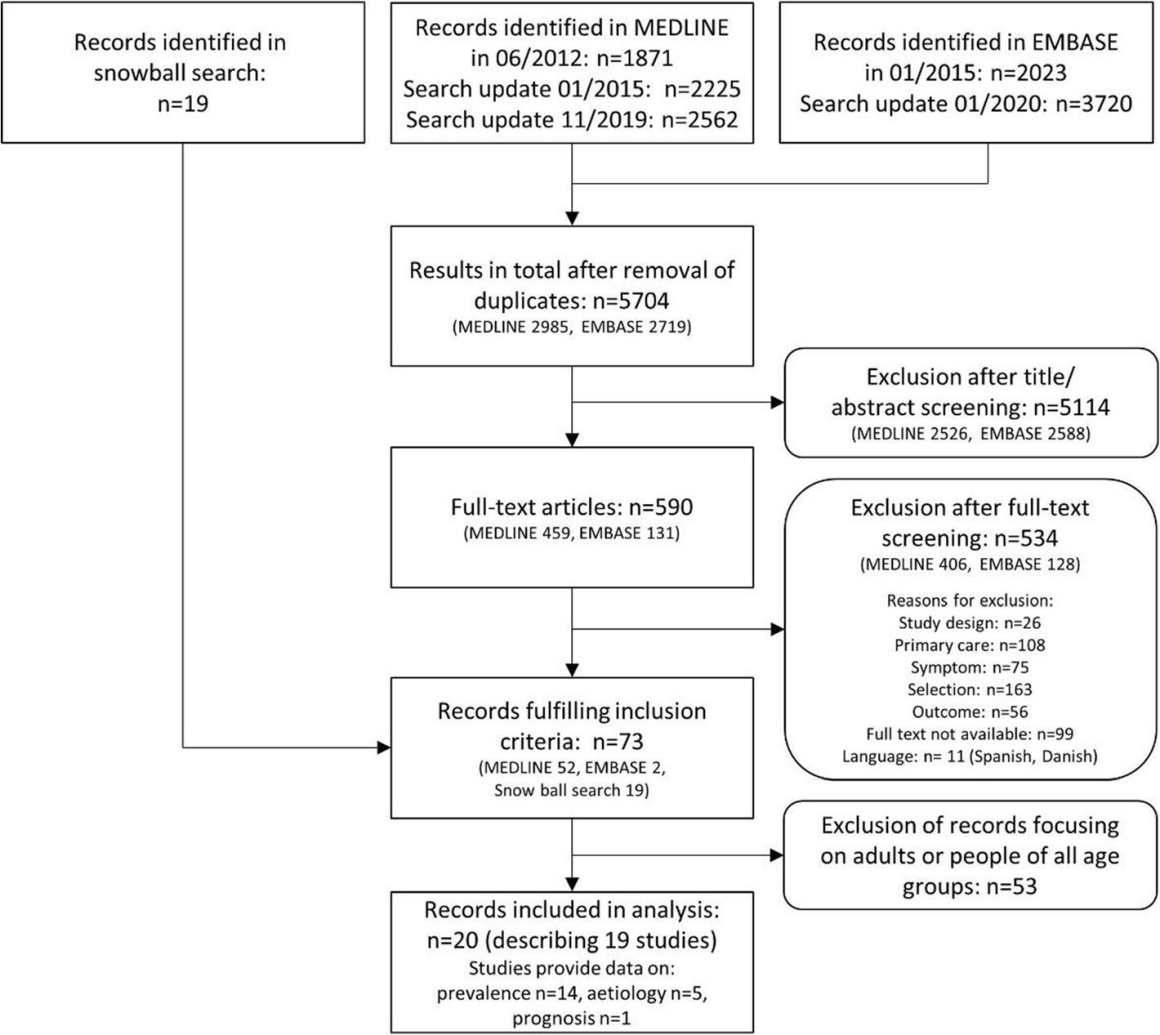
Search flow

We identified 5704 records (2985 in MEDLINE and 2719 in EMBASE) after removal of duplicates, plus 19 records from snowballing. Seventy-three papers fulfilled our inclusion criteria after full text screening; only 19 of these focused on children. Of the 19 studies, 14 provided data on the prevalence of cough, and five on underlying aetiologies. Only one of these studies reported on prognostic outcomes (see Fig. 1).

### Included studies

Most studies were conducted in Europe (*n* = 10), followed by North America (*n* = 3), Australia (n = 1), Africa (*n* = 4), and Asia (n = 1). Studies were published between 1971 and 2019. Mostly, data was assessed prospectively. The study populations consisted of 121 to 5100 patients, 188 to 92,888 consultations, 1196 to 70,489 reasons for encounters and 3371 episodes of care. Female patients ranged from 45 to 54%, and the mean age varied from 18.4 months to 9.8 years. Only three studies included children of all age groups (one study including some adults consulting paediatric offices). Solely children < 5/< 6/< 7 years were included in five studies, solely children ≥5 years in two studies. Nine studies excluded children > 11/> 14/> 15 years of age. Data was accrued by 1–209 primary care paediatricians or GPs in 1–57 paediatric or general practices. Further details on the included studies are given in Table 1.

**Table 1:** Description of the included studies

Studies	Country	Setting	Time of recruit-ment	Data assess-ment	Study population: Number of females	Age in study sample (years^1^)	Inclusion (IN) / Exclusion (EX) criteria	Out-come
Boyce 2019 [20]	Malawi	57 health facilities with 250 health surveillance assistants for integrated community case management	n.r.	prospectively	987 children ♀52%	Ø23.4 months	IN: first 4 children, aged 2–59 months, presenting to the health surveillance assistants for an initial consultation of their current illness EX: severely ill children who needed urgent referral to a health facility	Pre
Cazzato 2001 [21]	Italy	35 family paediatricians in Southern Italy	04–06/1998	prospectively	9917 children ♀50%	< 12: ≤2: 40.5% 3–6: 33.5% 7–12: 26%	IN: every patient-doctor contact on an index-day of the week over a 3-month period	Pre
Giannattasio 2014 [22]	Italy	3 primary care paediatric practices in Naples	12/2011–01/2012	prospectively	284 patients 188 consultations due to symptoms ♀ 54%	Ø 4.8 0–2: 25% 3–5: 36% 6–8: 20% 9–11: 13% 12–14: 6%	IN: all children aged 0–14 years observed in the index days	Pre
Hall 2017 [23]	Australia	1 Aboriginal-owned and operated comprehensive primary health-care service	02/2013–10/2015	prospectively	121 children ♀ 49%	0: 32.8% 1: 26.7% 2: 16.1% 3–4: 24.4% Ø18.4 months	IN: children presenting for any reason, aged < 5 years, registered at the healthcare service and parent willing/able to complete study requirements EX: family was planning to move from the area in the following 12 months	Pre
Harnden 2006 [24]	UK	18 general practices	10/2001–05/2005	prospectively	172 patients ♀ 45%	Ø 9.1 (positive pertussis serology) – 9.8 (negative pertussis serology)	IN: children, aged 5–16 years, with cough ≥14 days EX: refused blood sample	Aet Prog
Krishnan 2019 [25]	USA	1 predominantly suburban, academic paediatric faculty practice	1 year	retrospectively	560 consultations ♀ 47%	19 days - 18 years Ø 6.6 < 2: 18% 2–5: 41%	IN: children with completed electronic health record cough template	Aet
Leconte 2011 [26]	Belgium	36 primary care practices	02–03/2006	prospectively	345 patients	n.r.	IN: all consulting children aged 5–17 years	Pre
Mash 2012 [27]	South Africa	83 primary care clinics, 17 mobile clinics, 12 community health centres; nurse-led with support from doctors	1 year	prospectively	5545 reasons for encounter	< 1–14	IN: all ambulatory patients aged 0–14 years seen by health workers	Pre
Molony 2016 [28]	Ireland	1 large general practice with 4 GPs in a primary healthcare centre in North Cork	10/2010–10/2014	retrospectively	5100 patients 52,572 consultations 70,489 RFE	n.r.	IN: doctor-patient face-to-face encounters (children aged < 7 years) on all working days and 146 non-working days with a documentation of diagnostic code in the electronic medical record EX: contacts with practice nurse/ practice’s administrative team, telephone or ‘out-of-hours’ contacts	Pre
Morrell 1971/1972 [29, 30]	UK	1 general practice with 3 GPs	1 year	prospectively	707 patients 4467 consultations ♀ 51.3%	n.r.	IN: new patient-initiated consultations with symptoms not presented to any doctor in the previous 12 months, children aged 0–14 years EX: doctor-initiated consultations	Pre
Movsowitz 1987 [31]	South Africa	1 private paediatric practice in Cape Town	1984–1985	prospectively	256 patients	3 months −15 years	IN: patients with cough > 3 weeks	Aet
NAMCS Schappert 1999 [32]	USA	195 office-based paediatricians	01/1995–12/1996	prospectively	92,888 consultations ♀ 49.5%	< 15: 89.6% 15–24 6.2% 25–44: 2.5% 45–64: 1.1%	IN: office visits to non-federally employed paediatricians occurring during a randomly assigned 1-week reporting period EX: telephone contacts and visits made outside the physician’s office, visits to government-operated facilities and hospital-based outpatient departments	Pre
Nizami 1997 [33]	Pakistan	65 GPs and 29 paediatricians in Karachi	04–12/1992	prospectively	2433 consultations	n.r.	IN: children aged < 5 years	Pre
Njalsson 1992 [34]	Iceland	12 rural and 4 urban primary care health centres	01–12/1988	prospectively	67,746 RFE	0–14	IN: all contacts with children aged 0–14 years, including prescriptions, follow-up visits, tests, procedures and administrative visits	Pre
SESAM 2 Study Frese 2011 [35]	Germany	209 GPs in the federal state of Saxony	10/1999–09/2000	prospectively	805 patients 1196 RFE	0–4: 13.3% 5–9: 14.7% 10–14: 20.8% 15–19: 51.2%	IN: randomly selected children, aged 0- ≤ 19 years, presenting in general practice (tenth consultation of the consultation hour) previously known to the practitioner EX: house calls, patients already included in SESAM 2 study	Pre
Simoes 1997 [36]	Ethiopia	3 primary health centres with 6 outpatient clinic nurses	3 weeks in August	prospectively	449 patients ♀ 54%	2–11 months: 36%	IN: any sick child, aged 2 months – 5 years, presenting during study hours	Pre
TRANSITION Okkes 2002 [37]	Netherlands	54 family physicians in 23 locations in the Netherlands	1985–1995	prospectively	3371 episodes of care	n.r.	IN: episode data for all face-to-face encounters with paediatricians’ listed patients, aged 0–14 years, including encounters for prevention	Aet
Usherwood 1991 [38]	UK	1 general practice in Scotland	12/1986–01/1988	prospectively	466 consultations (including 147 home visits)	n.r.	IN: all health centre consultations of children, aged 2–13 years	Pre
Vinson 1993 [39]	USA, Canada	44 primary care practices in the Ambulatory Sentinel Practice Network (ASPN)	10/1990–01/1991	prospectively	1398 patients ♀ 47%	infancy - ≤14 Ø 4,8	IN: children aged 0–14 years with cough ≤1 month	Aet

Legend: ^1^ = unless otherwise stated, aet = aetiology of the symptom cough in primary care, n.r. = not reported, pre = prevalence of the symptom cough in primary care, prog = prognosis of the symptom cough in primary care, resp. = respectively, RFE = reasons for encounter, ♀ = female, Ø = mean

### Assessment of methodical quality and risk of bias

We found a high risk of substantial variation/clinical heterogeneity in the majority of studies (*n* = 11), mostly because certain age groups were excluded (Domain A). The risk of selection bias of patients was low, high and unclear in about a third of studies each. Concerning data collection (Domain B), most studies had a low risk of bias (*n* = 13), none a high risk. The risk of bias in diagnostic work-up (Domain C) was high in three studies, low in one and unclear in another. There was only one study with prognostic outcomes, showing a low risk of bias in prognostic work-up (domain D). Only five studies showed an overall low risk of bias (in all relevant domains). For details please see Additional File 3 and Additional File 4.

### Prevalence

**Fig. 2:**
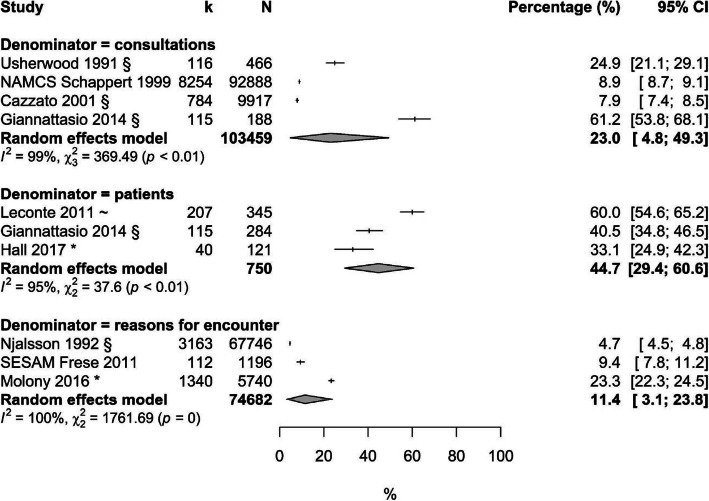
Prevalence of cough in children consulting in primary care of Western countries, sorted by denominators. Legend: * = study included solely children 5–17 years, CI = confidence interval, k = number of consultations because of a cough / reasons for encounter because of a cough / patients in consultation for a cough, N = total number of consultations / reasons for encounter / patients in consultation

Fourteen studies commented on prevalence or incidence of cough; five of these had an overall low risk of bias [21, 26, 27, 32, 35] (Additional File 4). Five studies describe the number of consultations for cough in relation to all consultations (about 24%). In these, patients consulting their physician repeatedly are counted more than once. This is different to the four studies which found about 35% of all patients seen in consultation complain about cough (in these studies each patient is counted only once). About 11% of all reasons for encounter (including physician consultations as well as consultations for prescriptions, follow-up visits, tests, procedures and administrative visits [34]) refer to the symptom cough. (Additional File 5) Figure 2 visualizes the respective prevalences in Western countries. Seasonal effects can be seen in studies recruiting solely in the European winter season; these show high estimates [22, 26], while studies taking place in Italian spring/summer and Ethiopian August show low estimates [21, 36]. Studies on older children show comparably low prevalences [32, 35]. Morrell et al. found a one-year cough rate of 267 (male) resp. 238 (female) per 1000 patients at risk (0–4 years) and 113 (female) resp. 160 (male) for children aged 5–14 years [30]. Age subgroup analyses didn’t minimize the high heterogeneity across studies.

Because only one study showed a low overall risk of bias and a low concern of clinical heterogeneity a subgrouping by quality was not possible [35].

### Aetiologies

Five studies presented prevalence data on aetiology [24, 25, 31, 37, 39]. Except for Harnden 2006 [24], who excluded children < 5 years, the studies included all age groups. As outcomes referred to different durations of cough, we omitted meta-analysis and presented the data descriptively (see Table 2). The most frequent aetiology for acute cough is upper respiratory tract infection, followed by bronchitis, and, for subacute and chronic cough, the most frequent aetiology is recurrent respiratory tract infection, asthma and pertussis. Estimates of frequencies are lower when related to episodes of care (Transition [37]) where several consultations for the same reason are summarized and counted only once, compared to consultations (Krishnan [25]), when patients may be counted several times. There is a high prevalence of pertussis in children coughing for more than two weeks, confirmed by serological evidence [24]. In all other studies aetiologies based on GPs’ working diagnoses [31, 37, 39] or on the diagnostic work-up were unclear [25], and attended by a high or unclear risk of bias. No study presented with an overall low risk of bias (Domains A, B, and C).

**Table 2:** Prevalences of selected aetiologies, referring to children in consultation for a cough in primary care / paediatric practices sorted by duration of cough

Study Study population	Vinson 1993 1398 patients	TRANSITION Okkes 2002 3371 episodes of care	Krishnan 2019 560 consultations	Harnden 2006 172 patients	Movsowitz 1987 256 patients
Duration of cough Aetiology	acute	all durations of cough		subacute/chronic	
	≤1 month			≥2 weeks	> 3 weeks
Upper respiratory tract infection	*n* = 873 62.4% [59.8; 65] viral: 35% (*n* = 494) bacterial: 27% (*n* = 379)	*n* = 1294 38.4% [36.7; 40.1]	*n* = 241 43% [38.9; 47.3]	n.r.	*n* = 71 27.7% [22.4; 33.7] (recurrent upper respiratory tract infection including bronchiolitis and bronchopneumonia)
Asthma	n = 129 9.2% [7.8; 10.9}	*n* = 100 3% [2.4; 3.6]	*n* = 101 18% [15; 21.5]	n.r.	n = 129 50.4% [44.1; 56.7]
Pertussis	n.r.	*n* = 34 1% [0.7; 1.4]	n.r.	*n* = 64 37,2% [30.1; 44.9]	*n* = 56 21.9% [17.1; 27.5]
Bronchitis / bronchiolitis	*n* = 465 33.3% [30.8; 35.6]	*n* = 757 22.5% [21.1; 23.9] (acute bronchitis / bronchiolitis)	*n* = 28 5% [3.4; 7.2]	n.r.	n.r.
Pharyngitis	n.r.	n.r.	*n* = 45 8% [6; 10.7]	n.r.	n.r.
Sinusitis	n.r.	*n* = 55 1.6% [1.2; 2.1]	n = 45 8% [6; 10.7]	n.r.	n.r.
Laryngitis / tracheitis	n.r.	*n* = 245 7.3% [6.4; 8.2]	n.r.	n.r.	n.r.
Croup	*n* = 30 2.1% [1.5; 3.1]	n.r.	n = 45 8% [6; 10.7%]	n.r.	n.r.
Pneumonia	*n* = 78 5.6% [4.5; 6.9]	*n* = 73 2.2% [1.7; 2.7]	*n* = 39 7% [5.1; 9.5]	n.r.	n.r.
Influenza	n.r.	*n* = 43 1.3% [0.9; 1.7]	n.r.	n.r.	n.r.
Otitis	n.r.	*n* = 42 1.2% [0.9; 1.7]	n = 28 5% [3.4; 7.2]	n.r.	n.r.
Other allergic diseases	*n* = 52 3.7% [2.8; 4.9]	n.r.	n.r.	n.r.	n.r.
Tonsillitis	n.r.	*n* = 54 1.6% [1.2; 2.1]	n.r.	n.r.	n.r.
Hypertrophy tonsils / adenoids	n.r.	*n* = 44 1.3% [1.0; 1.8]	n.r.	n.r.	n.r.
Tuberculosis	n.r.	n.r.	n.r.	n.r.	n = 1 0.4% [0; 2.5]
Bronchiectasis following pertussis	n.r.	n.r.	n.r.	n.r.	n = 1 0.4% [0; 2.5]
Persistently atelectatic right middle lobe	n.r.	n.r.	n.r.	n.r.	n = 1 0.4% [0; 2.5]
COPD	n.r.	n = 8 0.2% [0.1; 0.5]	n.r.	n.r.	n.r.
Heart failure	n.r.	*n* = 0 0% [0; 0.1%]	n.r.	n.r.	n.r.
Psychogenic cough	n.r.	n.r.	n.r.	n.r.	n = 0 0% [0; 1.8%]
Cystic fibrosis	n.r.	n.r.	n.r.	n.r.	n = 0 0% [0; 1.8%]
Foreign body nose / larynx / bronchus	n.r.	n.r.	n.r.	n.r.	n = 0 0% [0; 1.8%]

Legend: Every cell of table contains the absolute values (n), frequencies (%) and confidence interval [] of the study population with the respective aetiology. COPD = chronic obstructive pulmonary disease, n.r. = not reported

### Prognosis

Only one study reported prognostic outcomes. Harnden et al. recruited, from 18 practices in the United Kingdom, 179 children aged 5 to 16 years who had been coughing for 14 days or more [24]. Participants completed a daily cough diary for two weeks, then a weekly diary for the duration of the cough. The total duration of cough ranged from 24 to 192 days (the median duration was 112 days/resp. 58 days for patients with a positive/resp. negative pertussis serology). After two months, 62.3% of children were still coughing (positive pertussis serology: 85%, negative pertussis serology: 49%).

## Discussion

### Summary

Our systematic review identified 19 eligible studies. Prevalence estimates in Western countries varied widely between 12% of all reasons for encounter and up to 45% of patients consulting their physician. We found differential effects with lower prevalences in summer and in older children. Acute cough is mostly caused by infectious diseases like upper respiratory tract infection (RTI) or bronchitis; in chronic cough the most important diagnoses are RTI, asthma, and pertussis. Potentially serious diseases like pneumonia or tuberculosis are scarce. Duration of cough varies widely; spontaneous relief within a short time seems unlikely in subacute/chronic cough, with 62.3% of children still coughing after two months.

### Strengths and limitations

Sources of potential bias in systematic reviews are (1) criteria affecting the internal validity of studies (imprecise inclusion criteria and incomplete recruitment of study population), (2) limitations to the external validity of studies (setting characteristics and recruitment practice compromising the generalizability and applicability of the results), (3) methodological factors affecting the review’s internal validity (accuracy in literature search, screening process and data analysis), and (4) limitations to the review’s external validity [16, 17, 40].

To control the internal validity (1), we performed a substantial search and stated clear inclusion and exclusion criteria, but we omitted specialised paediatric journals or the term” paediatric practice” in our syntax. Still, we expect the misclassification to be low due to the comprehensive search of primary care settings including primary care paediatricians. As for the external validity (2) we did a double reviewer screening. Selection bias was minimized by considering only unselected study populations: In case of missing data regarding eligibility criteria we contacted study authors, although in some cases uncertainty remained. For (3) we performed a strict and standardized assessment of methodical quality, clinical heterogeneity and risk of bias [12]. Given the small number of included studies, we didn’t control for risk of bias across studies. However, publication bias seems unlikely, since there is no reason why prevalences, aetiologies or prognosis wouldn’t be published.

We found substantial methodological and clinical heterogeneity across age groups, study settings, healthcare systems, duration of cough, outcomes and reference parameters, which limits the external validity of our review (4). Cultural variables or gatekeeping influence the threshold to consulting a doctor, which is why we included only studies which had recruited in primary care settings. Still, age distribution in study samples may affect results: in German general practices over 50% of the study population were 15–19 years of age [35], while in two Italian family paediatricians’ offices 61–73% of children were < 6 years [21, 22]. In fact, the impact of cultural variables seems to be low, since heterogeneity was not minimized by age-related subgroups. The biggest limit to our study probably is the scarcity of high quality studies.

### Comparison with existing literature

Indeed, reviews report coughing as one of the most common reasons for consultation in routine paediatric and family practice [2, 41]. The majority of children experience 5 to 8 episodes of one week of cough throughout the year [41]. However, these studies are mostly based on secondary/tertiary care data [42, 43] or are population-based [44]. Age influences the development of the respiratory system in general [45], which explains the change of prevalences over lifetime, and distinctive age-related patterns [44], as shown in our study, with the lowest cough prevalences mainly in studies on older children (51.2% of children aged 15 to 19 years [35]). This is in accordance with the guidelines of the American College of Chest Physicians, who set the cut-off age for applying adult protocols at 14 years of age [11, 46].

The distinction between acute, subacute and chronic cough differs from what is applied in adults [46–49]. The US and Australian-New Zealand guidelines define acute cough in children to last < 2 weeks, subacute cough 2–4 weeks and chronic cough > 4 weeks. This is based on the natural course of upper RTI in children [9, 50] differing from the course in adults (< 3 weeks, 3–8 weeks and > 8 weeks) [7]. Triaging patients according the duration of cough is the first step in the diagnostic process, which is why aetiological data for both acute and chronic cough are required. However, the categorizations in the identified studies differed from those suggested in the cough guidelines [24, 31], which limits the impact of these studies for guideline development or validation.

Acute cough in children is mostly caused by upper RTI and bronchitis, which is confirmed by the current literature [2, 7, 51]. Its self-limiting course justifies a “wait-and-see” strategy, if no warning signs are present. Transient RTI is still a frequent cause of disease in chronic cough (despite the high share of prolonged courses as outlined above). Therefore, primary care guidelines recommend a 3–8 weeks’ observational period (as long as no signs of specific aetiologies are present) [7, 9]. In contrast to chronic cough in adults, the other two big causes of disease in children are asthma and pertussis. Their importance is confirmed by studies conducted on chronic cough in hospitals [42, 43, 52]. However, potential overdiagnosis and unnecessary long-term medication in children seem to occur frequently, since, especially in younger children, spirometry anti-asthma therapy trials are not sufficiently valid [7]. A frequency of 1 in 2 for asthma, as shown by Movsowith et al. (a study with a high risk of bias) should not tempt us to be less critical before initiating anti-asthma therapy for children. Instead, working with a category of “chronic non-specific persistent cough” is recommended as a more adequate way of facing the diagnostic uncertainty. Any cough in children with no signs of serious diseases can be summarized in this category [7, 9]. This supports primary care physicians in keeping awareness combined with regular re-evaluation, instead of jumping into hasty therapeutic processes. A more valid outcome is the high prevalence of pertussis found in a multicentre study in the UK with a low risk of bias [24]. In contrast to asthma, underdiagnosis of pertussis seems likely. This is especially relevant within the first 14 days, since antibiotics can reduce spread and school exclusion when given at an early stage of the disease – after that no specific treatment has been shown to be effective [7]. For evaluation of chronic cough, guidelines recommend relying on signs (“pointers”) in patient history or clinical examination for specific causes, like wheezing for asthma or a paroxysmal spasmodic cough for pertussis-like illness [7, 9]. Still, we need more aetiological evidence, because the diagnostic gain of all signs depends on setting specific pre-test probabilities.

We know from secondary care studies that acute cough caused by upper RTI lasts about 5.18 days (follow-up 6 days) in children [53]. In the primary care setting, acute cough seems to resolve in half of children within one week, and in 10–20% of children by three weeks [51, 54]. The methodological quality of these studies is low [51, 54]. Terms like “acute cough”, “acute bronchitis” or “chest infection” are often used simultaneously for different signs and symptoms [55]. To improve evidence regarding a wait-and-see strategy or observation phase authors advocate for more prognostic studies in primary care based on symptoms [54, 55], with a sufficiently long follow-up period and an unselected patient population.

## Conclusions

The prevalence of cough is higher in younger children than in adolescents, and lowest in summer. The high prevalence of upper RTI as an underlying disease and the low prevalence of potential serious illnesses seems to justify a “wait and see” approach to acute cough. Evidence on prolonged cough is scarce, but the prevalence of asthma and pertussis seems to rise substantially in subacute or chronic cough. Pertussis is especially prone to underdiagnosis. Other serious diseases like pneumonia or tuberculosis have a prevalence rate of less than 0.5%. There is hardly any data on prognosis of cough of children. Accordingly, thorough history taking and clinical examinations are mandatory to distinguish among differential diagnoses in coughing children. Further clarification of aetiological prevalences is needed to assess pre-test probabilities and the diagnostic gain from clinical signs which, once found to be valid, should be part of the standardized evaluation of cough.

## Supplementary Information

**Additional file 1.** PRISMA Checklist.

**Additional file 2.** Search strategy. Detailed search strategy.

**Additional file 3.** Tool for assessment of methodical quality, risk of bias and clinical heterogeneity. Instrument used to assess the quality of the studies and the risk of bias.

**Additional file 4.** Quality, risk of bias and clinical heterogeneity of included studies. Quality review results for all included studies.

**Additional file 5.** Prevalence / incidence of cough of children consulting in primary care (all studies). Forrest plots of prevalences and incidences of cough related to all studies that provided information on this.

## Data Availability

All data analysed during this study are drawn from published articles. The respective references and extracted numbers are all included in this article and its supplementary data files.

## Abbreviations

GP: General Practitioner
RTI: Respiratory tract infection
