# Supplementary material for: Coughing children in family practice and primary care: a systematic review of prevalence, aetiology and prognosis
Source: BMC Pediatr. 2021 Jun 4;21:260. doi: 10.1186/s12887-021-02739-4 (PMC8176681; doi:10.1186/s12887-021-02739-4)
Supplement: Supplementary file 2 — Additional file 2. Search strategy. Detailed search strategy. [file 12887_2021_2739_MOESM2_ESM.pdf]

Coughing Children in Family Practice and Primary Care: A systematic review of prevalence, aetiology and prognosis

Authors: Milena Bergmann,[a] Jörg Haasenritter,[a] Dominik Beidatsch,[a] Sonja Schwarm,[a] Kaja Hörner,[a] Stefan Bösner,[a] Paula Grevenrath,[a] Laura Schmidt,[a] Annika Viniol,[a] Norbert Donner-Banzhoff[a] and Annette Becker[a*]

[a] Department of General Practice/Family Medicine, University of Marburg, Marburg, Germany

[*] Corresponding author:
Annette Becker
Department of General Practice/Family Medicine, University of Marburg
Karl-von-Frisch-Str. 4, 35043 Marburg, Germany
E-mail: Annette.Becker@staff.uni-marburg.de

Additional File 2:

Title: Search strategy

	Syntax element	Syntax in MEDLINE	Syntax in EMBASE
Cough	Cough in all possible wordings in title and/or abstract	cough*[TIAB] OR<br>whoop*[TIAB] OR<br>expectorat*[TIAB]	cough*.ti,ab. or<br>whoop*.ti,ab. or<br>expectorat*.ti,ab.
		OR	or
	Cough as Mesh term	"Cough"[Mesh]	exp coughing
		AND	and
Primary Care	Term ‘primary care’ in all possible wordings in title and/or abstract	"general practitioner” [TIAB] OR<br>“general practitioners”[TIAB] OR<br>“general practice” [TIAB] OR<br>“family practice”[TIAB] OR<br>“family practitioners” [TIAB] OR<br>“family practitioner” [TIAB] OR<br>“family medicine” [TIAB] OR<br>“family physician” [TIAB] OR<br>“family physicians” [TIAB] OR<br>“family doctor” [TIAB] OR<br>“family doctors” [TIAB] OR<br>“primary care” [TIAB] OR<br>“family practices" [TIAB] OR<br>“GP” [TIAB] OR<br>“GPs” [TIAB] OR<br>“GPs’” [TIAB] OR<br>“GP’s” [TIAB]	(general adj1 practitioner).ti,ab. or<br>(general adj1 practitioners).ti,ab. or<br>(general adj1 practice).ti,ab. or<br>(family adj1 practice).ti,ab. or<br>(family adj1 practitioners).ti,ab. or<br>(family adj1 practitioner).ti,ab. or<br>(family adj1 medicine).ti,ab. or<br>(family adj1 physician).ti,ab. or<br>(family adj1 physicians).ti,ab. or<br>(family adj1 doctor).ti,ab. or<br>(family adj1 doctors).ti,ab. or<br>(primary adj1 care).ti,ab. or<br>(family adj1 practices).ti,ab. or<br>GP.ti,ab. or<br>GPs.ti,ab. or<br>GP's.ti,ab.
		OR	or
	Journal representing our research area	"BMC Fam Pract"[TA] OR<br>"Fam Pract"[TA] OR<br>"J Fam Pract"[TA] OR<br>"Fam Pract Res J"[TA] OR<br>"J Am Board Fam Pract"[TA] OR<br>"Br j gen pract"[TA] OR<br>“J R Coll Gen Pract” [TA] OR<br>“J Coll Gen Pract” [TA] OR<br>“J Coll Gen Pract Res Newsl”[TA] OR<br>"Can fam physician"[TA] OR<br>"Ann Fam Med"[TA] OR<br>"Aust fam physician"[TA] OR<br>"Scand J Prim Health Care"[TA] OR<br>"Eur J Gen Pract"[TA] OR<br>"Archives of family medicine"[Journal] OR<br>“J Gen Intern Med”[TA] OR<br>"Atencion primaria / Sociedad Española de Medicina de Familia y Comunitaria"[Journal]	bmc family practice.jn. or<br>family practice.jn. or<br>journal of family practice.jn. or<br>journal of the american board of family medicine.jn. or<br>british journal of general practice.jn. or<br>journal of the royal college of general practitioners.jn. or<br>canadian family physician.jn. or<br>annals of family medicine.jn. or<br>australian family physician.jn. or<br>scandinavian journal of primary health care.jn. or<br>european journal of general practice.jn. or<br>archives of family medicine.jn. or<br>journal of general internal medicine.jn.
		OR	
	Term ‘Primary Care’ (different wordings) in mailing address or name of institute of the author	“general practice” [AD] OR<br>“family practice*” [AD] OR<br>“family medicine” [AD] OR<br>“primary care” [AD] OR<br>community [AD]	-
		OR	or
	Primary Care as Mesh term	"General Practitioners"[Mesh] OR<br>"Family Practice"[Mesh] OR<br>"Physicians, Family"[Mesh] OR<br>"Primary Health Care"[Mesh] OR<br>"Physicians, Primary Care"[Mesh] OR<br>"General Practice"[Mesh] OR<br>“Community Health Services”[Mesh] OR<br>“Rural Health Services”[Mesh] OR<br>“Rural Health”[Mesh]	exp general practitioner/ or<br>exp family medicine/ or<br>exp primary medical care/ or<br>exp general practice/

		NOT	not
Limits	Limits to publications	editorial[pt] OR Addresses[pt] OR Bibliography[pt] OR Biography[pt] OR “Case Reports”[pt] OR Comment[pt] OR Dictionary[pt] OR Directory[pt] OR Festschrift[pt] OR “Government Publications”[pt] OR “Historical Article”[pt] OR “In Vitro”[pt] OR “Interactive Tutorial”[pt] OR Interview[pt] OR “Introductory Journal Article”[pt] OR Lectures[pt] OR “Legal Cases”[pt] OR Legislation[pt] OR News[pt] OR “Patient Education Handout”[pt] OR Portraits[pt] OR Webcasts[pt]	limit X to (book or book series or editorial or erratum or review)
		AND	and
	Limits to languages	Dutch[lang] OR English[lang] OR French[lang] OR German[lang] OR Italian[lang] OR Russian[lang] OR Spanish[lang] OR Swedish[lang]	limit X to (Dutch or English or French or German or Italian or Russian or Spanish or Swedish)
