# Supplementary material for: Coughing children in family practice and primary care: a systematic review of prevalence, aetiology and prognosis
Source: BMC Pediatr. 2021 Jun 4;21:260. doi: 10.1186/s12887-021-02739-4 (PMC8176681; doi:10.1186/s12887-021-02739-4)
Supplement: Supplementary file 3 — Additional file 3. Tool for assessment of methodical quality, risk of bias and clinical heterogeneity. Instrument used to assess the quality of the studies and the risk of bias. [file 12887_2021_2739_MOESM3_ESM.pdf]

Coughing Children in Family Practice and Primary care: A systematic review of prevalence, aetiology and prognosis

Authors: Milena Bergmann.[a] Jörg Haasenritter,[a] Dominik Beidatsch,[a] Sonja Schwarm,[a] Kaja Hörner,[a] Stefan Bösner,[a] Paula Grevenrath,[a] Laura Schmidt,[a] Annika Viniol,[a] Norbert Donner-Banzhoff,[a]and Annette Becker,[a*]

[a] Department of General Practice/Family Medicine, University of Marburg, Marburg, Germany

[*] Corresponding author:
Annette Becker
Department of General Practice/Family Medicine, University of Marburg
Karl-von-Frisch-Str. 4, 35043 Marburg, Germany
E-mail: Annette.Becker@staff.uni-marburg.de

Additional File 3

Title: Tool for assessing method quality, risk of bias and clinical heterogeneity

Legend: For each domain, reviewers independently answered the standardized key questions (yes, no or unclear) and assessed the risk of bias for the respective domain (low, high or unclear). In Domain A reviewers also rated their concern that the selection of patients and/or of GPs may have introduced substantial variation/ clinical heterogeneity (low, high or unclear).
Domain A and B referred to all studies regardless of the respective study outcome. Domain C was only considered if the respective study reported data on the underlying aetiologies of cough patients. Domain D was only considered if the respective study presented prognostic outcomes. In Domain C and D, key questions had to be answered separately for each diagnostic or prognostic category respectively.

**Domain A: Selection of patients and GPs (refers to all studies regardless the review question)**	
Item 1	Was the symptom to be investigated clearly described?
Item 2	Were the selection criteria of the patients clearly described?
Item 3	Was a consecutive or random sample of patients enrolled?
Item 4	Was it a multi-centre study?
Judgement: Risk that the selection of patients introduced bias: low, unclear, high	
Item 5	Did the selection criteria of the patients permit the study population to represent the full spectrum of those presenting with the symptom in the respective setting/ addressed in the review question?
Item 6	Were the participating health care professionals/ institutions representative for setting to be investigated in the review.
Judgement: Concern that the selection of patients and GPs introduced substantial variation or clinical heterogeneity: low, unclear, high	
**Domain B: Data collection and patient flow (refers to all studies regardless of the review question)**	
Item 7	Were data about the symptom und the inclusion criteria collected directly from the patients (as opposed to a proxy like a register, routine documentation)
Item 8	Was the same mode of data collection used for all patients?
Item 9	Was the number of non-responders/ dropouts unlikely to affect the results?
Judgement: Risk that the mode of data collection and/ or patient flow introduced bias: low, unclear, high	
**Domain C: Determination of the underlying aetiology/ diagnostic work-up (refers only to review question “What are the underlying conditions and their respective frequencies (differential diagnosis)?”). Had to be answered for each diagnostic category separately.**	
Item 10	Was the aetiologic category clearly defined?
Item 11	Was the diagnostic work up likely to correctly classify the respective aetiology?
Item 12	Did every patient receive the same diagnostic work up to detect the respective aetiology?
Judgement: Risk that the diagnostic work up introduce bias: low, unclear, high	
**Domain D: Determination of the prognosis/ prognostic work-up (refers only to review question “What is the prognosis of patients with the respective symptom presenting in the respective setting?”) Had to be answered for each prognostic category separately.**	
Item 13	Was the prognostic outcome clearly defined?
Item 14	Did the study design include a comparison group without the symptom?
Item 15	Was the work up/ measurement likely to correctly classify the respective prognostic outcome?
Item 16	Did every patient receive the same work up/ mode of data collection to verify the respective prognostic outcome?
Judgement: Risk that the prognostic work up introduce bias: low, unclear, high	
