# Supplementary material for: Coughing children in family practice and primary care: a systematic review of prevalence, aetiology and prognosis
Source: BMC Pediatr. 2021 Jun 4;21:260. doi: 10.1186/s12887-021-02739-4 (PMC8176681; doi:10.1186/s12887-021-02739-4)
Supplement: Supplementary file 4 — Additional file 4. Quality, risk of bias and clinical heterogeneity of included studies. Quality review results for all included studies. [file 12887_2021_2739_MOESM4_ESM.pdf]

Coughing Children in Family Practice and Primary Care: A systematic review of prevalence, aetiology and prognosis

Authors: Milena Bergmann,[a] Jörg Haasenritter,[a] Dominik Beidatsch,[a] Sonja Schwarm,[a] Kaja Hörner,[a] Stefan Bösner,[a] Paula Grevenrath,[a] Laura Schmidt,[a] Annika Viniol,[a] Norbert Donner-Banzhoff[a] and Annette Becker[a*]

[a] Department of General Practice/Family Medicine, University of Marburg, Marburg, Germany

[*] Corresponding author:
Annette Becker
Department of General Practice/Family Medicine, University of Marburg
Karl-von-Frisch-Str. 4, 35043 Marburg, Germany
E-mail: Annette.Becker@staff.uni-marburg.de

Additional File 4:

Title: Quality, risk of bias and clinical heterogeneity of included studies

Domain	A: **Selection of patients and GPs** (assessed for all included studies)								B: **Data collection and patient flow** (assessed for all included studies)				C: **Determination of the underlying aetiology of the symptom** (assessed for 4 studies including aetiologic outcomes)				D: **Determination of the prognosis** (assessed for 1 study including prognostic outcomes)				
Item	1	2	3	4		5	6		7	8	9		10	11	12		13	14	15	16	
+ Yes<br>- No<br>? Unclear<br><br>Studies	Was the symptom to be investigated clearly described?	Were the selection criteria of the patients clearly described?	Was a consecutive or random sample of patients enrolled?	Was it a multi-centre study?	Risk that the selection of patients introduced bias	Did the selection criteria of the patients permit the study population to represent the full spectrum of those presenting with the symptom in the respective setting/ addressed in the review question?	Were the participating health care professionals/ institutions representative for setting to be investigated in the review?	Concern that the selection of patients and GPs introduced substantial variation or clinical heterogeneity	Were data about the symptom und the inclusion criteria collected directly from the patients (as opposed to a proxy like a register, routine documentation)?	Was the same mode of data collection used for all patients?	Was the number of non-responders/ dropouts unlikely to affect the results?	Risk that the mode of data collection and/ or patient flow introduced bias	Was the aetiologic category clearly defined?	Was the diagnostic work up likely to correctly classify the respective aetiology?	Did every patient receive the same diagnostic work up to detect the respective aetiology?	Risk that the diagnostic work up introduce bias	Was the prognostic outcome clearly defined?	Did the study design include a comparison group without the symptom?	Was the work up/ measurement likely to correctly classify the respective prognostic outcome?	Did every patient receive the same work up/ mode of data collection to verify the respective prognostic outcome?	Risk that the prognostic work up introduce bias
Boyce 2019	-	-	?	+	?	-	-	high	+	+	+	low									
Cazzato 2001	-	?	+	+	low	-	+	high	+	+	+	low									
Giannattasio 2014	-	?	+	-	?	+	+	low	+	+	+	low									
Hall 2017	-	+	?	-	high	-	+	high	+	+	+	low									
Harnden 2006	+	-	?	?	?	-	+	high	+	+	?	?	+	+	+	low	+	-	+	+	low
Krishnan 2019	-	+	?	-	high	+	+	low	-	?	?	?	-	?	?	?					
Leconte 2011	+	-	+	+	low	?	+	?	+	+	+	low									
Mash 2012	-	?	+	+	low	+	-	high	+	+	+	low									
Molony 2016	-	+	+	-	high	-	+	high	-	+	+	?									
Morrell 1971/1972	-	+	?	-	high	-	?	high	+	+	?	?									
Movsowitz 1987	+	-	?	-	high	+	+	low	-	?	+	?	?/-*	?	-	high					
NAMCS Schappert 1999	-	?	+	+	low	-	+	high	+	+	+	low									
Nizami 1997	-	-	?	-	high	-	?	high	+	+	+	low									
Njalsson 1992	-	-	?	+	?	+	+	low	-	+	+	?									
SESAM 2 Study Frese 2011	-	+	+	+	low	+	+	low	+	+	+	low									
TRANSITION Okkes 2002	-	?	+	+	low	+	+	low	+	+	?	low	-	?	-	high					
Simoes 1997	-	-	+	-	?	-	-	high	+	+	+	low									
Usherwood 1991	-	?	+	-	high	-	+	high	+	+	+	low									
Vinson 1993	+	-	+	+	low	+	+	low	+	+	?	low	?/-*	?	-	high					

*=varying assessments for different aetiologic categories
