# Supplementary material for: Coughing children in family practice and primary care: a systematic review of prevalence, aetiology and prognosis
Source: BMC Pediatr. 2021 Jun 4;21:260. doi: 10.1186/s12887-021-02739-4 (PMC8176681; doi:10.1186/s12887-021-02739-4)
Supplement: Supplementary file 5 — Additional file 5. Prevalence / incidence of cough of children consulting in primary care (all studies). Forrest plots of prevalences and incidences of cough related to all studies that provided information on this. [file 12887_2021_2739_MOESM5_ESM.pdf]

Coughing Children in Family Practice and Primary Care: A systematic review of prevalence, aetiology and prognosis

Authors: Milena Bergmann,[a] Jörg Haasenritter,[a] Dominik Beidatsch,[a] Sonja Schwarm,[a] Kaja Hörner,[a] Stefan Bösner,[a] Paula Grevenrath,[a] Laura Schmidt,[a] Annika Viniol,[a] Norbert Donner-Banzhoff[a] and Annette Becker[a*]

[a] Department of General Practice/Family Medicine, University of Marburg, Marburg, Germany

[*] Correspondng author:
Annette Becker
Department of General Practice/Family Medicine, University of Marburg
Karl-von-Frisch-Str. 4, 35043 Marburg, Germany
E-mail: Annette.Becker@staff.uni-marburg.de

Additional File 5:

Title:

Prevalence / incidence of cough of children consulting in primary care (all studies)

Legend:

* = study included solely children <5/<6/<7 years, § = study included solely children <12/<14/<15 years, ~ = study included solely children 5-17 years, CI = confidence interval, k = number of consultations because of a cough / (incidental) reasons for encounter because of a cough / patients in consultation for a cough, N = total number of consultations / (incidental) reasons for encounter / patients in consultation

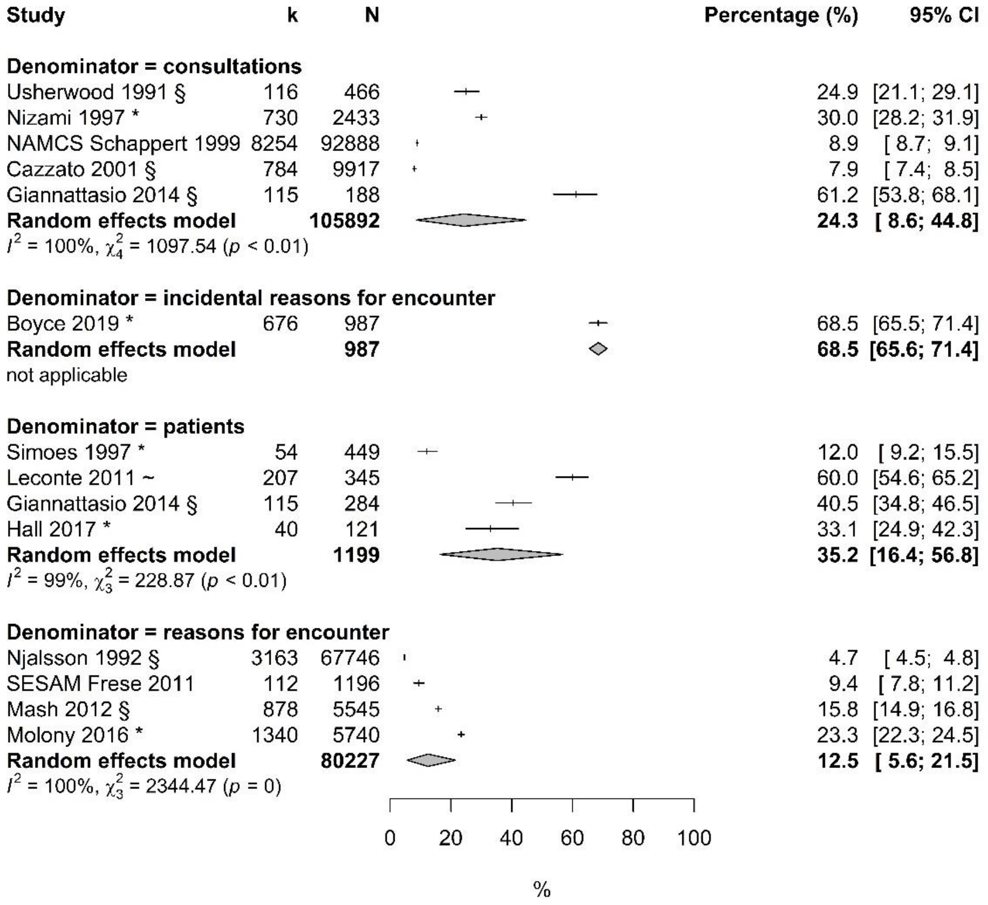
Study k N Percentage (%) 95% CI
Denominator = consultations
Usherwood 1991 § 116 466 24.9 [21.1; 29.1]
Nizami 1997 * 730 2433 30.0 [28.2; 31.9]
NAMCS Schappert 1999 8254 92888 8.9 [8.7; 9.1]
Cazzato 2001 § 784 9917 7.9 [7.4; 8.5]
Giannattasio 2014 § 115 188 61.2 [53.8; 68.1]
Random effects model 105892 24.3 [8.6; 44.8]
I^2 = 100%, χ^2_4 = 1097.54 (p < 0.01)
Denominator = incidental reasons for encounter
Boyce 2019 * 676 987 68.5 [65.5; 71.4]
Random effects model 987 68.5 [65.6; 71.4]
not applicable
Denominator = patients
Simoes 1997 * 54 449 12.0 [9.2; 15.5]
Leconte 2011 ~ 207 345 60.0 [54.6; 65.2]
Giannattasio 2014 § 115 284 40.5 [34.8; 46.5]
Hall 2017 * 40 121 33.1 [24.9; 42.3]
Random effects model 1199 35.2 [16.4; 56.8]
I^2 = 99%, χ^2_3 = 228.87 (p < 0.01)
Denominator = reasons for encounter
Njalsson 1992 § 3163 67746 4.7 [4.5; 4.8]
SESAM Frese 2011 112 1196 9.4 [7.8; 11.2]
Mash 2012 § 878 5545 15.8 [14.9; 16.8]
Molony 2016 * 1340 5740 23.3 [22.3; 24.5]
Random effects model 80227 12.5 [5.6; 21.5]
I^2 = 100%, χ^2_3 = 2344.47 (p = 0)
0 20 40 60 80 100
%
